# WHAT IS THE EPIDEMIOLOGICAL PROFILE OF ACUTE HAND INFECTIONS AT A HOSPITAL IN SAO PAULO?

**DOI:** 10.1590/1413-785220243201e277229

**Published:** 2024-05-06

**Authors:** Thomas Yi Teh Lee, Lucas Alves Nemer, Alessandro Ayres Vianna, Yussef Ali Abdouni, Fabrício Luz Cardoso, Antonio Carlos da Costa

**Affiliations:** 1. Irmandade Santa Casa de Misericordia de Sao Paulo, Department of Orthopedics e Traumatology “Pavilhao Fernandinho Simonsen,” Sao Paulo, SP, Brazil.; 2. Irmandade Santa Casa de Misericordia de Sao Paulo, Department of Orthopedics e Traumatology “Pavilhao Fernandinho Simonsen,” Hand and Microsurgery Group, Sao Paulo, SP, Brazil.

**Keywords:** Bacterial Infections, Hands, Epidemiology, Anti-Bacterial Agents, Orthopedics, Infecções Bacterianas, Mãos, Epidemiologia, Antibacterianos, Ortopedia

## Abstract

**Objective:**

To determine the epidemiological profile of patients treated at a philanthropic hospital specialized in Orthopedics and Traumatology, located in a significant urban center, and evaluate the efficacy of initial empirical antibiotic treatment.

**Methods:**

Patients diagnosed with hand infections from September 2020 to September 2022 were included, excluding cases related to open fractures or post-surgical infections and those with incomplete medical records. The chi-square test was performed using STATISTICA ^®^ software to correlate various variables.

**Results:**

A total of 34 patients participated, including 24 men and 10 women, with an average age of 41.9 years. Most male patients had Diabetes Mellitus, HIV, and drug addiction, and they resided in urban areas. Half of the patients did not report any apparent trauma. The most common infectious agent was Staphylococcus aureus*. Nearly 62% of patients required a change in the initial antibiotic regimen, with Penicillin being the most frequently substituted medication. Beta-lactam antibiotics and Quinolones were the most effective.

**Conclusion:**

These results suggest the importance of carefully evaluating the epidemiological profile of patients with acute hand infections and improving initial empirical treatment to ensure appropriate and effective therapy. ***Level of Evidence IV, Cross-Sectional Observational Study.***

## RESUMO

**Objetivo:**

*Determinar o perfil epidemiológico de pacientes atendidos em um hospital filantrópico referência em Ortopedia e Traumatologia localizado em um centro urbano importante, e avaliar a eficácia do tratamento antibiótico empírico inicial.*

**Métodos:**

*Foram incluídos pacientes com diagnóstico de infecção na mão, atendidos no período de setembro de 2020 a setembro de 2022, excluindo-se casos relacionados a fraturas expostas ou pós-cirúrgicas e aqueles com prontuários incompletos. Realizou-se o teste do qui-quadrado, utilizando o software STATISTICA* ^®^ *para correlacionar diversas variáveis.*

**Resultados:**

*Participaram do estudo 34 pacientes, sendo 24 homens e 10 mulheres, com média de idade de 41,9 anos. A maioria era de homens, com alta incidência de diabetes mellitus, HIV e drogadição, que residiam em áreas livres. Metade não relatou trauma evidente. O agente infeccioso mais comum foi o Staphylococcus aureus. Aproximadamente 62% dos pacientes precisaram de troca do esquema inicial de antibióticos, sendo a penicilina o medicamento mais frequentemente substituído. Os antibióticos beta-lactâmicos e quinolonas foram os mais eficientes.*

**Conclusão:**

*Esses resultados sugerem a importância de avaliar cuidadosamente o perfil epidemiológico dos pacientes com infecções agudas na mão e aprimorar o tratamento empírico inicial para garantir uma terapia adequada e eficaz.* ***Nível de Evidência IV, Estudo Observacional Transversal.***

## INTRODUCTION

 Hand infections are common diagnoses in orthopedic clinical practice. ^1^ They represent 1/3 of the patients admitted to hand surgery services, and it is estimated that 60% of hand infections are posterior to trauma, of which 30% result from human or animal bites and 10% from other causes, such as drug injection. ^2^ Most patients seek the health services due to pain, which is commonly associated with redness, edema, heat, and erythema. ^3^ Pain is most often the initial symptom, followed by edema, redness and local warmth. ^3^

 Acute hand infections usually begin due to a breach of the skin integrity as result of an unnoticed injury. Diabetes mellitus, immunosuppression, malnutrition, alcoholism, and drug abuse are risk factors for the emergence of hand infection. ^4^ The patient’s age is also an important risk factor for hand conditions. ^4^ Most infections result from injuries in the home or workplace and involve Gram-positive bacteria. *Staphylococcus aureus* is the most frequently isolated microorganism found in hand infections; it is identified in 44–80% of cases in other studies. ^5^^-^^7^ There has been an increase in the incidence of methicillin-resistant *Staphylococcus aureus* (MRSA) in the last 15 years, with its incidence estimated at about 65%. ^8^^,^^9^

It is believed that the distribution of different infections is related to the epidemiological profile of the population cared for in a specific service. This study seeks to establish the epidemiological profile of patients with acute hand infections who are treated in a reference philanthropic hospital of Orthopedics and Traumatology located in a major urban center. The other purpose of this research is to evaluate the effectiveness of initial empirical antibiotic treatment.

## METHODOLOGY

 This is a cross-sectional observational study of epidemiological data of patients with acute hand infections, who attended the previously described service, from September 2020 to September 2022. The study was approved by the Research Ethics Committee under opinion No. 5,818,523 and all patients signed an informed consent form. The data were collected via medical records, with relevant information being transferred to an Excel ^®^ file. 

The inclusion criteria were: hand infection diagnoses. Infections after an open or post-surgical fracture were excluded, as were patients with incomplete or inaccurate medical records.

Regarding epidemiological data of the patients, gender, occupation, comorbidities, history of alcoholism or drug use, housing conditions (public area or not) and trauma mechanisms were evaluated; when applicable, age was also evaluated. For age, patients were allocated into three groups: three to 34 years (nine subjects), 35 to 47 years (12 subjects) and 50 to 68 years (13 subjects). Infections were classified according to the affected tissue and site. Type of trauma, when present, was also a criterion of investigation.

For infection characterization, the following data were retrieved: infectious agent, exam results of C-reactive protein (CRP) and erythrocyte sedimentation rate (ESR), as well as the antibiotic applied at the entrance, and failures that led to medication change.

 Lastly, the chi-square test was carried out using the STATISTICA ^®^ software for the following correlations: drug use *versus* mechanism of trauma; alcohol use *versus* mechanism of trauma; age *versus* microorganisms isolation; living and working conditions *versus* microorganisms isolation; drug use *versus* isolation of microorganisms; diabetes mellitus *versus* microorganisms isolation; alcohol use *versus* microorganisms isolation; mechanism of trauma *versus* microorganisms isolation; age *versus* effectiveness of the initial antibiotic; living conditions *versus* effectiveness of the initial antibiotic; drug use *versus* effectiveness of the initial antibiotic; diabetes mellitus *versus* effectiveness of the initial antibiotic; alcohol use *versus* effectiveness of the initial antibiotic; mechanism of trauma *versus* effectiveness of the initial antibiotic; microorganisms isolation *versus* effectiveness of the initial antibiotic and initial antibiotic *versus* effectiveness of the initial antibiotic. 

## RESULTS

 A total of 34 patients met the inclusion criteria, 24 men (70.6%) and 10 women (29.4%). No individuals were excluded. The mean age of these patients was 41.9 years, ranging from three to 68 years (standard deviation = 11.1). As for housing, 10 patients (29.4%) were residents of public areas (including four recyclers and six unemployed individuals). We also observed the presence of three retirees (8.9%), three students (8.9%) and two individuals with unspecified profession (5.8%) ( Table 1 ). 

**Table 1.** Occupational and housing conditions of the individuals evaluated by the study.

**Profession**	**Total**
Health worker	1
Retiree	3
Kitchen assistant	1
Business owner	3
Caretaker	1
Unemployed (public area resident)	6
Homemaker	1
Electrician	1
Student	3
Vendor of farm products	1
Plasterer	1
Valet	1
Unspecified	2
Construction worker	1
Painter	1
Door attendant	1
Recycler (public area resident)	4
Security guard	1
Computer technician	1
**Overall Total**	**34**

**Figure 1.**
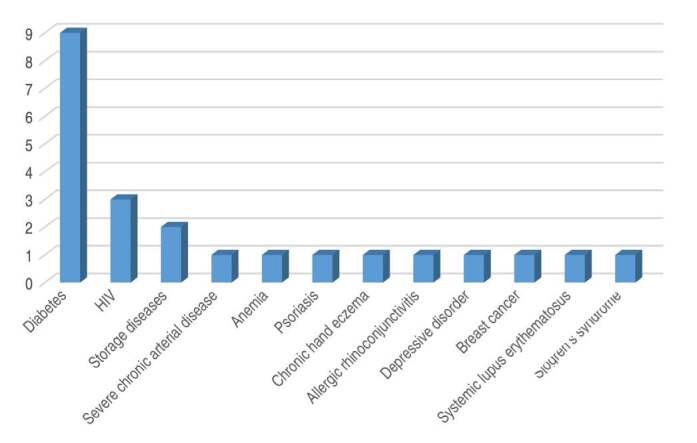
Comorbidities presented in order of prevalence.

Figure 1 lists the comorbidities presented by these patients. The most common comorbidity was diabetes mellitus, affecting nine patients (26.5%); HIV was the second most prevalent, in three patients (8.9%). Regarding habits, 17 patients reported drug use (50%) and 11 patients reported being alcohol users (32.4%). 

**Figure 2.**
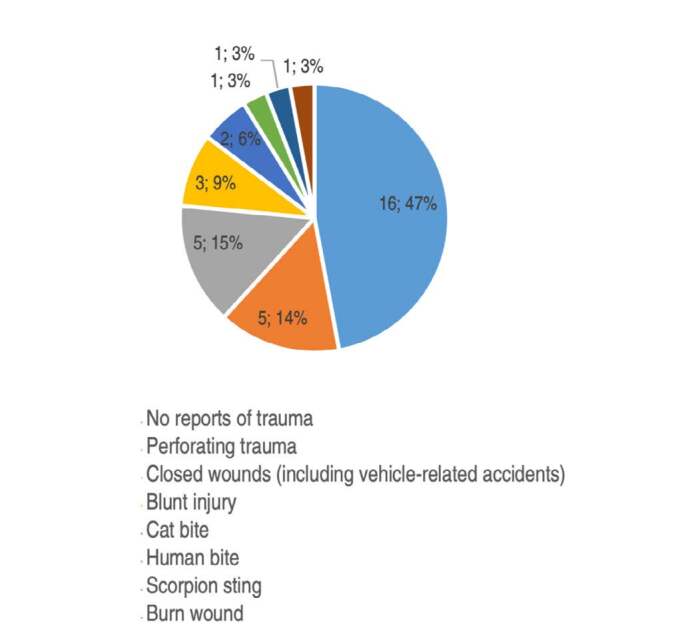
Mechanism of trauma in order of prevalence.

 Among the 34 evaluated patients, 47% reported no previous trauma, 14.7% reported blunt trauma and 14.7% had perforating trauma. Human and cat bites accounted for 9% of cases each, totaling three cases. The other mechanisms of trauma are shown in Figure 2 . 

 The most frequent hand infections according to Table 2 were: 12 cases of flexor tenosynovitis (32.4%) and four cases of extensor tenosynovitis (10.8%). Table 2 also show the other diagnoses. 

**Table 2.** Description of the diagnosis and site of infection of the evaluated patients.

**Diagnosis**	**Number**	**Percentage**
Abscess on the distal phalanx	1	2.7%
Abscess on the intermediate phalanx	2	5.4%
Abscess on the proximal phalanx	2	5.4%
Abscess in the first commissure	1	2.7%
Abscess in the dorsal wrist	1	2.7%
Abscess in the dorsal metacarpophalangeal region	1	2.7%
Abscess in the thenar region	2	5.4%
Abscess on the volar zone of the thumb	2	5.4%
Metacarpophalangeal cellulitis	1	2.7%
Subungual abscess	1	2.7%
Distal phalanx necrosis	2	5.4%
Distal phalanx necrosis + ink-machinery-related accident/foreign-body	1	2.7%
Distal phalanx osteomyelitis /paronychia	1	2.7%
Distal interphalangeal pyoarthritis – human bite	1	2.7%
Proximal interphalangeal pyoarthritis – cat bite	1	2.7%
Extensor tenosynovitis	4	10.8%
Flexor tenosynovitis	12	32.4%

*Staphylococci* species were the most prevalent, with *S. aureus* occurring in 26.5% of cases and coagulase-negative in one (3%). Cultures were negative in 53% of cases. 

In laboratory analysis, it was observed that 28 patients (82.4%) presented changes in CRP, with values ≥ 1, ranging from 1 to 36.7 (standard deviation = 13.82), while 24 (70.6%) demonstrated changes in ESR, with values above 13 for men and 20 for women, ranging from 4 to 120 (standard deviation = 36.26). Exam collection was not possible for two patients (5.9%).

The following significant relationships were obtained: (1) drug use versus the need for change in antibiotic; (2) infectious agent versus need for change in antibiotic; and (3) initial antibiotic class versus need for change in antibiotic. The results revealed significant associations between the variables analyzed and the need to change antibiotic. In the case of drug use, the total chi-square (39.20) was higher than the critical value (3.841), indicating a relevant association between this variable and the need to change antibiotics in the studied group. Similarly, for the infectious agent, the total chi-square (9.5152) was higher than the critical value (7.815), demonstrating a significant association between the type of infectious agent and the need for change in antibiotic. Furthermore, in relation to the initial antibiotic class, the total chi-square (100.5273) was higher than the critical value (12.592), indicating a strong association between this variable and the need to change antibiotics in the analyzed group.

For the other associations, no statistical significance was found; the age group that most needed change in antibiotic was those aged 50 to 68 years (61.5%), with eight patients who needed such change (standard deviation = 3.96), while in the age group from three to 34 years there was a need for change in 44.4% of patients (standard deviation = 8.81) and in the age group from 35 to 47 years in 25% of patients (standard deviation = 4.29).

## DISCUSSION

 Hand infections, when not properly identified and treated, can result in tissue damage, loss of function, and even permanent disability, leading to significant morbidity and mortality rates. ^10^

 In this study, we found a predominance of infections in male patients (70.6%), agreeing with what has been described in the literature in the past 20 years. ^4^

 Patient’s age is a key factor in hand infections. In our study, the ages ranged from the first decade of life to the sixth decade. According to Flevas et al. ^11^ older patients with degenerated vessels, poor perfusion, and weakened skin barrier are more likely to attend health services with infected upper limb conditions. To Gafur et al. ^12^ newborns are also prone to hand infections due to immature immune systems and sharp nails that can hurt their fingertips. 

 Patients with hand infections are often individuals who engage in handwork, such as farmers, animal breeders, bricklayers, fishers, and water sports athletes. ^10^^,^^11^^,^^13^ In addition, medical professionals such as dentists and surgeons are also susceptible to hand infections. ^10^^,^^11^ It is important to highlight that manual workers who do not have adequate hand protection—often due to financial condition—also face risks of infection due to poor-quality equipment. 

The health service where this study was conducted is in the central region of the city, close to areas with high population density and poor living conditions. Therefore, the studied sample corresponds to a socioeconomically vulnerable population.

 Malnutrition and poor hygiene were observed in a considerable proportion of patients, including those living on the streets. Moreover, we observed a high percentage of patients who were drug users (50%), surpassing the statistics found in the literature. ^5^ Notably, unlike the more common report of injectable drug use, such as that of Houshian, Seyedipour and Wedderkopp, ^5^ the main drug used by the population in this study was crack cocaine. The use of this substance is intricately linked to the manipulation of sharp objects during its consumption, increasing the risk of hand trauma. 

 In our study, we found that 47% of patients had no identified traumatic event, while bites represented only 9% of cases. Note that, there is a possibility of bias due to improper filling of medical records and the accuracy of information obtained from patients. However, in 14 cases (41.1%), an additional risk factor was identified, such as diabetes, HIV, or living in extremely poor areas, suggesting a possible protein-related malnutrition. Several studies report the adverse effects of malnutrition on wound healing and infection progression. ^14^^,^^15^

 According to previous studies, ^2^^,^^5^^,^^6^^,^^16^ the most common pathogens in hand infections are the *Staphylococcus aureus* and the coagulase-negative *Streptococci* . In our sample, we observed that *Staphylococci* were the most prevalent, with *S. aureus* present in 26.5% of cases, and only one case of coagulase-negative *Staphylococcus* (2,9%). We also found a high rate of negative cultures (53%), whilst the literature reports a range of 10–30%. ^2^^,^^5^^,^^6^^,^^16^

 The number of insufficient samples and inadequate culture methods, such as the use of agar-agar medium without investigating other agents that require specific means or insufficient evaluation time, may explain the high rate of missing results in this study, which might be a diagnostic bias, as previously indicated. ^17^ Due to our sample including a significant number of immunocompromised patients, there is a higher chance of atypical pathogens or the involvement of multiple agents, which require targeted investigation methods. This is highlighted by the fact that there was more than one case of associated infection with *Streptococcus viridans* and *Morganella morganii* . 

 Among the positive cultures of drug users, five had infections caused by *Staphylococcus aureus* (83.3%), while only one was caused by beta hemolytic *Streptococci* (16.7%). These findings are in accordance with Dastagir et al. ^16^ As aforementioned, this association is related to the type of drug used. In the literature, ^5^ injectable drugs have a greater association with the development of infections. In our sample, however, we observed a different pattern, as crack cocaine was the most reported drug. 

 A surprising finding in this study was a significant percentage of patients who required a change in the initially used antibiotics. The hospital protocol regarding the Infectious Diseases service recommends the intravenous administration of ciprofloxacin associated with oxacillin as empirical antibiotic therapy, immediately after culture collection. This protocol seeks to provide broad antibacterial coverage for both Gram-positive and Gram-negative germs. In 21 cases (61.8%), it was necessary to change antibiotic therapy based on the results of the cultures. As described by Intravia, Osterman and Tosti, ^17^ 12 patients (35.2%) who started treatment with ciprofloxacin and oxacillin, according to the recommended schedule, needed to change antibiotic therapy. It is relevant to discuss whether the high rate of negative cultures—with possible atypical agents—influenced this change, considering that the sample of the our study differs from the literature. 

 There is a positive correlation between CRP levels and the severity of the infection, as well as the extent of the affected areas, according to Rasnake and Dooley. ^18^ In our study, despite some patients’ malnourishment, most showed an initial increase in CRP and ESR values. However, due to the significant loss of follow-up, it was not possible to establish a correlation between clinical evolution and laboratory parameters. 

The statistical results revealed valuable information about the association between different categories of antibiotics and the need for changing the medicine. Penicillin, first generation cephalosporin, sulfonamide and diaminopyrimidines required more changes than other categories. Notably, among these four categories, penicillin was the least efficient. Also, in Gram-negative infections, followed by fungus and negative cultures were more associated with change in antibiotic.

On the other hand, beta-lactam and quinolone were the most effective, and we did not find a microorganism that needed fewer changes in antibiotic. However, it is essential to consider other clinical and individual factors in the choice of treatment, and the decision should be taken by a health professional based on all the particularities and needs of each patient.

 Finally, the age group that most needed a change in antibiotic was the 50 to 68 years old group. Although there was no significant relevance, there was a greater tendency to change antibiotics in this age group. To obtain more robust conclusions, it would be essential to have a larger sample. In Stromberg’s seminal work, ^19^ from 1989, it was suggested that, compared to younger populations, antibiotic sensitivity is consistently lower in older adults. This study was later corroborated by Heppner et al. ^20^ —some 20 years later—when they found that the prevalent microbiology of infection changes with aging, leading to differences in optimal antimicrobic protocol for severe infections. 

## CONCLUSION

 Patients with hand infections were mostly men, with a high incidence of diabetes mellitus, HIV and drug addiction, living in public areas. Almost half individuals did not report evident trauma. *Staphylococcus aureus* was the most common agent. In 61.8% of cases, change in the initial antibiotic regimen was necessary. Penicillin was the least efficient, while beta-lactam and quinolone were the most. Gram-negative patients needed more changes in antibiotics. 
